# Killer immunoglobulin-like receptor 2DS5 is associated with recovery from coronavirus disease 2019

**DOI:** 10.1186/s40635-021-00409-4

**Published:** 2021-09-03

**Authors:** Vadim Lesan, Moritz Bewarder, Carlos Metz, André Becker, Sebastian Mang, Evi Regitz, Lorenz Thurner, Frank Neumann, Igor Kos, Konstantinos Christofyllakis, Guy Danziger, Stephan Stilgenbauer, Robert Bals, Philipp M. Lepper, Dominic Kaddu-Mulindwa, Torben Rixecker

**Affiliations:** 1 Department of Internal Medicine I (Oncology, Hematology, Clinical Immunology, Rheumatology), Saarland University Medical Center, University Hospital, Saarland, 66421 Homburg, Germany; 2 Department of Internal Medicine V (Pneumology, Allergology and Critical Care Medicine), Interdisciplinary COVID-19 Center, University Hospital, Saarland, Homburg, Germany

**Keywords:** KIR, NK cells, SARS-CoV-2, COVID-19, ARDS, Intensive care unit

## Abstract

**Background:**

Despite numerous advances in the identification of risk factors for the development of severe coronavirus disease 2019 (COVID-19), factors that promote recovery from COVID-19 remain unknown. Natural killer (NK) cells provide innate immune defense against viral infections and are known to be activated during moderate and severe COVID-19. Killer immunoglobulin-like receptors (KIR) mediate NK cell cytotoxicity through recognition of an altered MHC-I expression on infected target cells. However, the influence of KIR genotype on outcome of patients with COVID-19 has not been investigated so far. We retrospectively analyzed the outcome associations of NK cell count and KIR genotype of patients with COVID-19 related severe ARDS treated on our tertiary intensive care unit (ICU) between February and June 2020 and validated our findings in an independent validation cohort of patients with moderate COVID-19 admitted to our tertiary medical center.

**Results:**

Median age of all patients in the discovery cohort (*n* = 16) was 61 years (range 50–71 years). All patients received invasive mechanical ventilation; 11 patients (68%) required extracorporeal membrane oxygenation (ECMO). Patients who recovered from COVID-19 had significantly higher median NK cell counts during the whole observational period compared to patients who died (121 cells/µL, range 16–602 cells/µL vs 81 cells/µL, range 6–227 cells/µL, *p*-value = 0.01). KIR2DS5 positivity was significantly associated with shorter time to recovery (21.6 ± 2.8 days vs. 44.6 ± 2.2 days, *p*-value = 0.01). KIR2DS5 positivity was significantly associated with freedom from transfer to ICU (0% vs 9%, *p*-value = 0.04) in the validation cohort which consisted of 65 patients with moderate COVID-19.

**Conclusion:**

NK cells and KIR genotype might have an impact on recovery from COVID-19.

**Supplementary Information:**

The online version contains supplementary material available at 10.1186/s40635-021-00409-4.

## Background

A novel coronavirus (SARS-Cov-2) causing coronavirus disease 2019 (COVID-19) was first identified in December 2019 in Wuhan, China, upon a series of pneumonias of unknown cause [1]. Since then, SARS-CoV-2 spread worldwide, reaching pandemic levels [2]. The clinical presentation of COVID-19 covers a broad spectrum, ranging from asymptomatic upper respiratory infections to severe acute respiratory distress syndrome (ARDS) [3]. Genome-wide association analyses in a large case–control study including healthy volunteers as a control group identified certain gene clusters to be associated with patients suffering from COVID-19 and requiring oxygen supplementation [4]. Yet, associations of individual genetic imprints with outcome in patients already suffering from moderate or severe COVID-19 are still missing.

Natural killer (NK) cells play a central role in the immune response against viral infections [5–8]. In patients with COVID-19, especially in those with severe symptoms, lower NK cell counts were consistently reported [9]. In contrast to peripheral blood, the human lung is enriched in NK cells during the course of COVID-19 [10]. Moreover, there has been evidence for an exhausted NK cell phenotype in COVID-19 [11]. Further characterization analyses identified distinct NK cell immunotypes to be associated with COVID-19 severity and an increase in adaptive NK cells in SARS-CoV-2-infected patients with severe disease [12]. Taking it all together, these data underline why NK cells might influence the course of COVID-19.

NK cells express a highly polymorphic group of killer immunoglobulin-like receptors (KIR), capable of binding specific human leukocyte antigens (HLA) in order to recognize an altered MHC-I expression, mostly HLA-C antigens on virus-infected target cells (missing-self principle) [13, 14]. Evidence linking specific KIR genotypes and susceptibility to viral infections has been broadly published [15, 16]. However, the influence of KIR genotype on the course of COVID-19 has not been investigated so far. Therefore, we aimed to investigate the associations of KIR genotypes and clinical course of COVID-19-related severe ARDS in patients treated on our tertiary intensive care unit (ICU) between February and June 2020 and validated our findings in an independent validation cohort of patients with moderate COVID-19 admitted to our tertiary medical center.

## Methods

### Patient characteristics and study design

In this retrospective study, we included all patients with confirmed severe COVID-19 who were treated on our ICU between February and June 2020 (discovery cohort) and all patients with moderate COVID-19 admitted to our tertiary medical center (validation cohort) of whom written informed consent by themselves or their next of kin was obtained. In the same time period, a total of 40 patients with COVID-19 were treated on our intensive care unit.

Severe COVID-19 was defined as polymerase chain reaction (PCR)-confirmed SARS-COV2 infection and severe ARDS as defined by the Berlin definition with a PaO_2_/FiO_2_ ratio of less than 100 mmHg (Horowitz Index (P/F ratio) < 100 mmHg) and bilateral pulmonal infiltration on radiological imaging [17]. Moderate COVID-19 was defined as PCR-confirmed SARS-COV2 infection and evidence of lower respiratory disease during clinical assessment or imaging and who have an oxygen saturation (SpO_2_) ≥ 94% on room air at sea level.

This study and post hoc analysis were part of the COORSAAR register study and approved by the local ethics committee (Ethikkommission der Ärztekammer des Saarlandes, Ethics approval number 62/20). The study was performed in accordance with the rules of the Declaration of Helsinki.

### Study endpoints

Study endpoint of this retrospective study for the discovery cohort was recovery from COVID-19 at day 28 after ICU admission. Patients were considered recovered if they were free from oxygen supplementation regardless of their hospitalization status.

Study endpoint for the validation cohort was freedom from transfer to ICU.

### KIR genotyping

We extracted genomic deoxyribonucleic acid (DNA) from whole blood samples obtained from patients using the Qiagen column-based method (QIAmp DNA Blood Mini Kid, Qiagen, Chatsworth, CA). We performed KIR genotyping by real-time polymerase chain reaction (RT-PCR) (BioRad CFX Connect Real-Time System) as previously described [18]. In brief, PCR reaction mixtures contained KIR-specific primers, internal control primers (GALC), patient DNA and SYBR Green I Master Mix (Applied Biosystems, Foster City, CA). Presence of the specific KIR was confirmed by analyzing individual melting temperatures. Seventeen KIR genes were analyzed in this study. KIR haplotype was discriminated according to existing nomenclature [19].

### Natural killer cells and laboratory parameters

Measurements of leucocytes were performed using a Sysmex XN-L TM automated hematology analyzer. Immune characterization of lymphocytes was performed by flow cytometry using a BD FACSCanto™ II cell analyzer. Flow cytometry was performed according to the “Guidelines for the use of flow cytometry and cell sorting in immunological studies” [20]. NK cell subpopulation was characterized using a Beckman–Coulter Navios TM Analyzer. Total counts of lymphocytes and NK cell subpopulation were therefore calculated using a dual-platform system. Tests were performed twice weekly until transfer to normal care unit or hospital discharge due to clinical improvement or death. Cellular parameters included lymphocytic differentiation into NK cells (CD56+, CD16+). Laboratory parameters included complete blood cell count, CRP and IL-6 and were also obtained at least twice weekly until transfer from ICU to normal care unit due to clinical improvement or death.

### Statistical analysis

Patient baseline characteristics were summarized descriptively according to data type. One-sample Kolmogorov–Smirnov test and one-sample Chi-square test were used to assess the distribution of data. Differences between groups were assessed with Chi-square and independent median test. All statistical tests were two-sided with a significance level of 5%. Adjustment for multiple comparison was performed in all presented data. Kaplan–Meier analysis was used to assess the differences in outcome between the groups. Log-rank pooled over strata was used as a comparing method. All data were analyzed using SPSS v25.0 (IBM, Ehningen, Germany).

## Results

### Severe COVID-19 discovery cohort

In the discovery cohort, we identified 16 patients with COVID-19-related severe ARDS and written informed consent obtained from themselves or their next of kin, treated on our intensive care unit between February and June 2020. Patient baseline (day 1 of ICU admission) characteristics are shown in Table 1. Median age of all patients was 61 years (range 50–71 years). All analyzed patients were Caucasian males. Main comorbidities were arterial hypertension (8/16, 50%) and diabetes mellitus type 2 (2/16, 12.5%). Pre-existing diseases were equally distributed between outcome groups (Table 1).

**Table 1:** Baseline clinical characteristics and laboratory parameters

Parameters	All patients (*n* = 16)	Dead (*n* = 6)	Recovered (*n* = 10)	*p*-value*
Median age in years, (IQR)	61 (8)	60.5 (15)	63 (7)	0.63
BMI > 30, %	4 (25)	1 (16.6)	3 (30)	0.56
Diabetes mellitus, %	2 (12.5)	1 (16.6)	1 (10)	0.70
Hypertension, %	8 (50)	3 (50)	5 (50)	–
Baseline Horowitz Index (P/F-ratio), median (IQR)	86.5 (25)	100 (22)	82 (28)	0.60
Baseline WBC count, median (range), cells/µL	9940 (6390–31,500)	9105 (8000–14,210)	12,020 (6390–31,500)	0.60
Baseline NK cell count, median (range), cells/µL	58 (6–236)	25 (6–60)	72.5 (16–236)	0.11
Baseline CRP, median (range), mg/L	226 (49–376)	235 (94–376)	218.5 (49–326)	0.60
Baseline IL-6, median (range), pg/mL	184 (11–2397)	116 (78–2397)	237 (11–1306)	0.60

*NK* natural killer cells, *IQR* interquartile range, *WBC* white blood count, *CRP* C-reactive protein, *BMI* body mass index, *IL-6* interleukin-6, *P/F-ratio* partial pressure/fractional inspired oxygen

*Based on independent median test and Pearson Chi-square test/Fisher’s test, corrected for multiple tests

### Patient outcome

Median time of stay on ICU ward was 21.5 days (range 11–47 days) (Table 2). All patients received invasive mechanical ventilation and 11 patients (68%) required extracorporeal membrane oxygenation (ECMO). Regarding clinical outcome, 6 (37.5%) patients died, and 10 (62.5%) patients fully recovered. The cause of death was COVID-19 related in all patients. Complications during ICU stay included pneumothorax in 3 (18%) patients, catheter-related sepsis in 2 (12%), acute mesenteric ischemia with compartment syndrome in 1 (6%) patient and aortic dissection in 1 (6%) patient. Median time to recovery from COVID-19 was 40.5 days (range 11–47 days). Median time to death was 21 days (range 15–39 days). Recovery at day 28 after admission was reached in 4/16 (25%) patients. Clinical characteristics, laboratory parameters, duration of ECMO therapy and Horowitz Index (P/F-ratio) were similar in patients who recovered at 28 days and those who did not (Additional file 1: Table S1).

**Table 2:** Clinical characteristics and laboratory parameters at the end of observation period

Parameter	All patients (***n*** = 16)	Dead (***n*** = 6)	Recovered (***n*** = 10)	***p***-value*
Median time of stay on ICU, days (range)	21.5 (11–47)	21 (15–39)	40.5 (11–47)	0.14
Median time to ECMO, days (range)	1 (0–29)	1 (1–12)	0 (0–29)	0.28
ECMO, Nr. (%)	11 (68)	6 (100)	5 (50)	0.09
Median NK-cell count during whole observational period (range), cells/µL	110 (6–602)	81 (6–227)	121 (16–602)	**0.01**
Median CRP level during whole observational period (range), mg/L	164 (6–462)	235 (43–423)	139 (6–642)	**0.01**
Median IL-6 levels during whole observational period (range), pg/mL	209 (23–3407)	614 (217–3407)	86 (23–2313)	**0.007**

*p*-values in bold reflect statistical significance at a level of *p*-value < 0.05

*ECMO* extracorporeal membrane oxygenation, *NK* natural-killer cells, *IQR* interquartile range, *CRP* C-reactive protein, *IL-6* interleukin-6, *ICU* intensive care unit

*Based on independent median test and Pearson Chi-square test/Fisher’s test, corrected for multiple tests

### Laboratory parameters

**Fig. 1:**
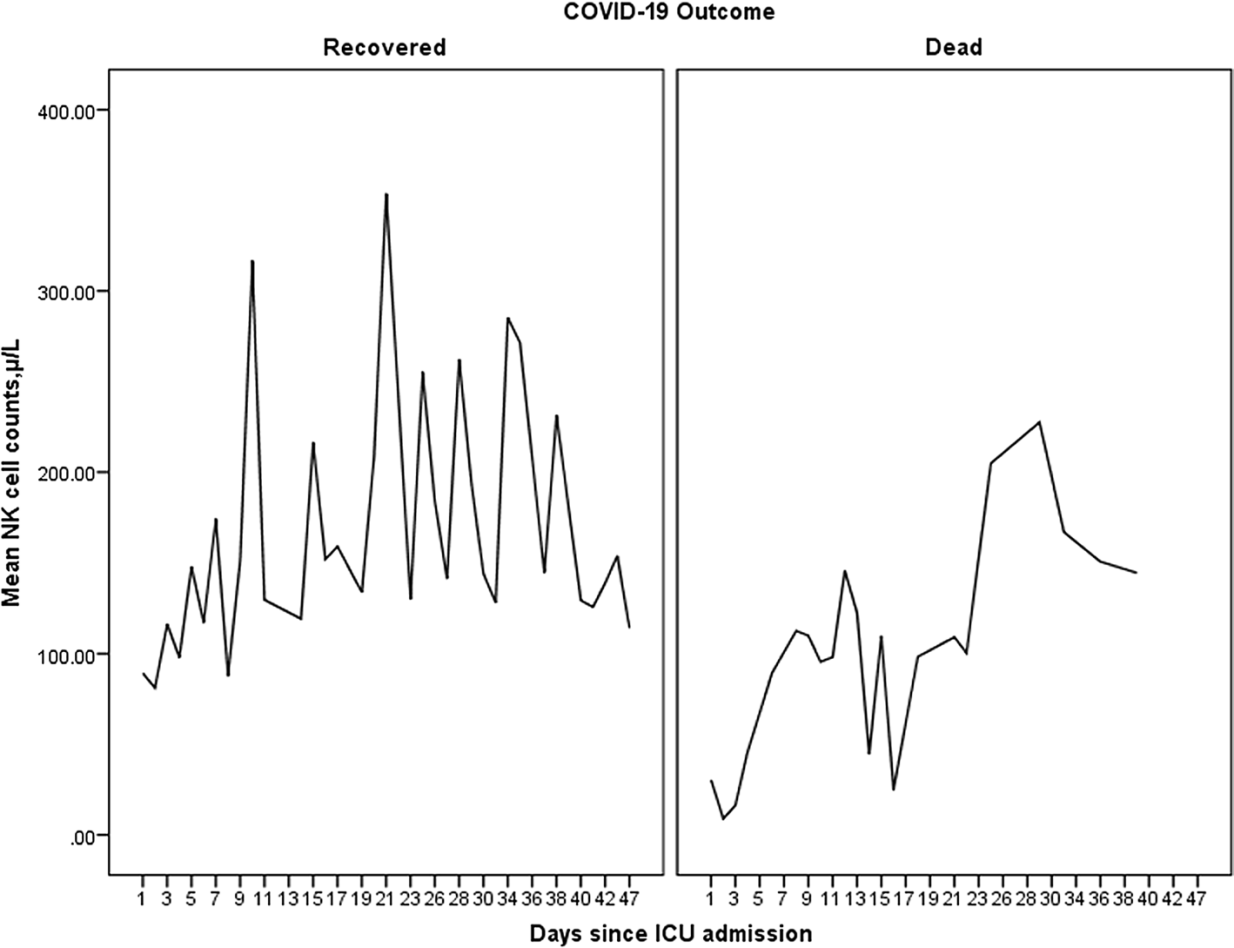
Mean natural killer cell count over whole observational period according to patient outcome. Outcome related mean natural-killer cell counts over whole observational period showing higher natural killer cell counts in patients who recovered from severe COVID-19 compared to patients who died

Baseline laboratory parameters are summarized in Table 1 and Additional file 1: Table S1. Median leukocyte count on admission was 9940 cells/µL (range 6390–31,500 cells/µL). Median natural-killer cell count on ICU admission was 58 cells/µL (range 6–236 cells/µL). Median baseline leukocyte count was comparable between outcome groups (recovered: 12,020 cells/µL, range 6390–31.500 cells/µL vs. dead: 9105 cells/µL, range 8000–14,210 cells/µL, *p*-value = 0.6). On admission, NK cell count was similar in patients who recovered and those who died (recovered: 72.5 cells/µL vs. 25 cells/µL, *p*-value = 0.11). However, patients who recovered showed significantly higher median NK cells counts during the whole observational period compared to patients who died (121 cells/µL, range 16–602 cells/µL vs. 81 cells/µL, range 6–227 cells/µL, *p*-value = 0.01) (Table 2 and Fig. 1). Furthermore, NK cell counts correlated negatively with disease severity (Pearson correlation − 0.31, *p*-value = 0.001) and C-reactive protein (CRP) levels (Pearson correlation − 0.31, *p*-value = 0.001). Patients who died of COVID-19 had significantly higher median CRP levels during whole observational period compared to patient who recovered (Table 2). IL-6 showed no significant differences at baseline between the two outcome groups (Table 1). However, during the whole observational period, patients who died had significantly higher IL-6 levels compared to patients who recovered (*p*-value = 0.007) (Table 2).

### KIR genotype and recovery

**Fig. 2:**
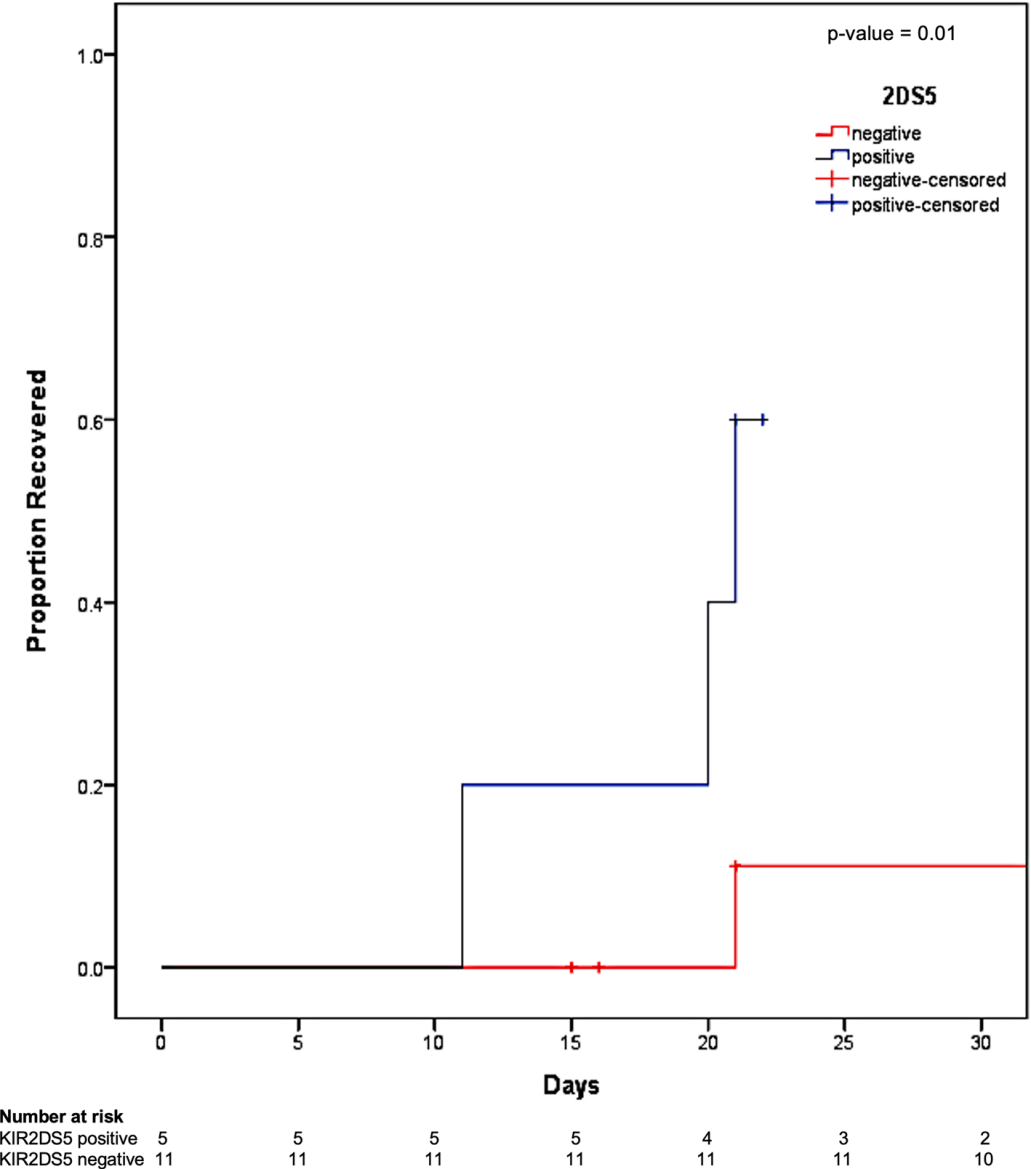
Kaplan–Meier estimates on recovery regarding KIR2DS5 status. Cumulative estimates on recovery from COVID-19-related severe ARDS showing significantly higher proportion of recovery in KIR2DS5-positive patients

KIR genotype distribution across all patients is presented in Additional file 1: Table S2. The frequency of different KIR genes of all patients suffering from COVID-19-related severe ARDS was comparable to existing data on KIR genotype distribution upon the general German Caucasian population (Additional file 1: Table S2). In 16 patients suffering from COVID-19-related severe ARDS, we did not observe a significant difference in single KIR and KIR haplotype distribution between patients who died and those who recovered (Additional file 1: Table S3). Hence, only KIR2DS5 was significantly associated with time to recovery by day 28 (Fig. 2 and Additional file 1: Figure S1). In particular, KIR2DS5-positive patients demonstrated shorter time to recovery compared to KIR2DS5-negative patients, respectively (mean 21.6 ± 2.8 days vs. mean 44.6 ± 2.2 days, *p*-value = 0.01). By day 28 after admission, 60% of KIR2DS5-positive patients had recovered compared to only 9% of KIR2DS5-negative patients (*p*-value = 0.01).

There was no significant difference between KIR2DS5-positive and negative patients regarding clinical and laboratory baseline characteristics (Additional file 1: Table S4). Furthermore, the rate of complications in KIR2DS5-positive and negative patients was comparable (Additional file 1: Table S4).

### Moderate COVID-19 validation cohort

To validate our findings from the discovery cohort, we determined the KIR2DS5 status in 65 Caucasian patients with moderate COVID-19 and written informed consent from themselves or their next of kin admitted to our tertiary medical center during the same time period. KIR2DS5 positivity was observed in 20 of 65 patients (30%). Median age was 68 years (IQR: 22 years) (Additional file 1: Table S5). Males represented 57% of all patients. 23 (35%) patients required oxygen supplementation. KIR2DS5-positive patients required less oxygen supplementation than KIR2DS5-negative patients, but without reaching statistical significance (22% vs. 46%, *p*-value = 0.08). Only 1 (5%) KIR2DS5-positive patient died of COVID-19, compared to 6 (14%) KIR2DS5-negative patients (*p*-value = 0.33). None of the KIR2DS5-positive patients required transfer to intensive care unit (ICU), compared to 9 (22%) KIR2DS5-negative patients (*p*-value = 0.04) (Additional file 1: Figure S2).

## Discussion

In this retrospective, single-center study, we analyzed the associations of KIR genotype with recovery of patients with COVID-19-associated severe ARDS and validated our findings in patients with moderate COVID-19. We identified KIR2DS5 positivity to be associated with improved clinical course in these patients.

All of our analyzed patients from the discovery cohort of patients with severe COVID-19 were males, which is in line with previous reports of COVID-19 showing consistently a male predominance regarding infection rates [21, 22]. Additionally, the comorbidity rates were comparable with previously published data [23]. Cumulative data from 18 studies, including 2984 COVID-19 patients, reported increased white blood cell and neutrophil counts and decreased lymphocyte, platelet and eosinophil counts in patients with severe forms of respiratory distress, emphasizing the cellular involvement in the course of COVID-19 [24]. Regarding NK cell counts, our results are consistent with previously published literature, showing reduced NK cell numbers in patients who died compared to those who survived COVID-19 [22]. Additionally, higher CRP and IL-6 levels were associated with adverse outcome, which is in line with previously published studies [25–27].

Our data show a comparable distribution between KIR genotype in patients with severe COVID-19 and the general German Caucasian population, suggesting, that the individual KIR status does not correlate with disease severity. Nevertheless, we found, that KIR2DS5 positivity was significantly associated with a superior course of disease in patients already suffering from moderate or severe COVID-19.

Although the ligand of KIR2DS5 remains unknown, activating KIR2DS5 was shown to have a protective role in different diseases, including acute rejection reaction of kidney grafts, malaria, human immunodeficiency virus infection and Hepatitis C-induced hepatocellular carcinoma [28]. The activation of KIR2DS5 triggers both, NK cell cytotoxicity and interferon γ (IFN‐γ) release [29]. This enhanced activation could explain the observed shorter mean time to recovery, since an exhaustion of NK cells was previously shown to be associated with inferior outcomes in patients suffering from COVID-19 patients [30]. Moreover, reduced expression of IFN-γ by NK cells in patients with severe disease as compared to patients with moderate disease was observed in a previous immunological characterization analysis of 21 patients with COVID-19 [31].

However, our study does have some limitations. First, it is a single-center retrospective study with a small sample size of patients even though it was performed during very challenging clinical circumstances of the pandemic-imposed limitations. Second, IFN‐γ levels of patients participating in this study were not available. Nevertheless, our results provide a first look into the possible role of KIR in the context of COVID-19 and suggest KIR2DS5 to promote disease recovery from moderate or severe COVID-19.

Although the number of patients included in our analysis does not allow for valid conclusions, association of both, stimulating KIR2DS5 and higher NK cell counts with recovery in patients with severe COVID-19 and validation of our findings in patients with moderate COVID-19 warrant further analyses regarding the impact of KIR genotype on the course of COVID-19.

## Conclusions

NK cell count and KIR genotype might influence the course COVID-19. Stimulating KIR2DS5 was associated with superior outcome in moderate or severe COVID-19.

## Supplementary Information

**Additional file 1: Table S1: **Clinical characteristics and laboratory parameters based on recovery by day 28 (discovery cohort). **Table S2: **KIR prevalence in 16 patients with COVID-19 related severe ARDS compared to the general German Caucasian population. **Table S3: **KIR genes and Haplotype regarding clinical outcome. **Figure S1.** Kaplan Meier Estimates on Recovery regarding KIR genotype. Cumulative estimates on recovery from COVID-19 related severe ARDS showing no statistically significant differences regarding specific KIR genotype. **Table S4. **Clinical characteristics and laboratory parameters regarding KIR2DS5 status. **Table S5. **Baseline characteristics of 65 patients with moderate COVID-19 (validation cohort) according to KIR2DS5 status. **Figure S2.** Kaplan Meier Estimates on transfer to Intensive Care Unit (ICU) of 65 patients with moderate COVID-19 (validation cohort) according to KIR2DS5 Status. Cumulative estimates on time to transfer to intensive care unit (ICU) showing statistical significant difference regarding KIR2DS5 status.

## Data Availability

The datasets generated during and/or analyzed during the current study are available from the corresponding author on reasonable request.

## Abbreviations

ARDS: Severe acute respiratory distress syndrome
COVID-19: Coronavirus disease 2019
CRP: C-reactive protein
DNA: Deoxyribonucleic acid
ECMO: Extracorporeal membrane oxygenation
HLA: Human leukocyte antigen
ICU: Intensive care unit
IFN‐γ: Interferon γ
IL-6: Interleukin-6
IMC: Intermediate care ward
KIR: Killer-immunoglobulin-like receptor
NK: Natural killer
PCR: Polymerase chain reaction
RT-PCR: Real-time polymerase chain reaction
SARS-Cov-2: Severe acute respiratory syndrome coronavirus type 2
