# Supplementary material for: Killer immunoglobulin-like receptor 2DS5 is associated with recovery from coronavirus disease 2019
Source: Intensive Care Med Exp. 2021 Sep 3;9:45. doi: 10.1186/s40635-021-00409-4 (PMC8412971; doi:10.1186/s40635-021-00409-4)

Additional File

Table 1: Clinical characteristics and laboratory parameters based on recovery by day 28 (discovery cohort)

| Parameters | all patients  (n =16) | recovered by day 28  (n = 4) | not recovered by day 28  (n = 12) | p-value* |
| --- | --- | --- | --- | --- |
| median Age in years, (IQR) | 61 (8) | 63 (13) | 60.5 (8) | - |
| baseline P/F ratio (IQR), mmHg | 86.5 (25) | 77 (51) | 86.5 (22) | 0.37 |
| invasive mechanical ventilation (%) | 16 (100) | 4 (100) | 12 (100) | - |
| ECMO (%) | 11 (68) | 2 (50) | 9 (75) | 0.36 |
| median Leucocyte count at baseline (IQR), cells/µl | 9940 (9670) | 15300 (19280) | 9800 (5760) | 0.56 |
| median lymphocyte count at baseline (IQR), cells/µl | 654 (600) | 816.5 (952) | 651 (538) | 1.0 |
| median NK-cell count at baseline (IQR), cells/µl | 58 (59) | 122 (161) | 45 (49) | 0.56 |
| median NK-cell count during whole observational period (IQR), cells/µl | 110 (95) | 122 (102) | 107 (100) | 0.50 |
| median CD4/CD8 ratio,  (IQR) | 4 (3) | 4.4 (10.4) | 4 (2.4) | 1.0 |

* Based on independent Median Test and Pearson Chi-square Test/Fisher’s Test and corrected for multiple tests

ECMO, Extracorporeal Membrane Oxygenation; NK, Natural-Killer Cells; IQR,interquartile range

Additional File

Table 2: KIR prevalence in 16 patients with COVID-19 related severe ARDS compared to the general German Caucasian population

| KIR  positive/all | severe COVID-19 | German Caucasian Population* | p-value** |
| --- | --- | --- | --- |
| 2DL1 | 16/16 (100%) | 181/199 (91%) | 0.21 |
| 2DL2 | 7/16 (43.75%) | 101/199 (51) | 0.57 |
| 2DL3 | 16/16 (100%) | 181/199 (91%) | 0.21 |
| 2DL4 | 16/16 (100%) | 99/99 (100%) | - |
| 2DL5 | 8/16(50%) | no data | - |
| 2DS1 | 5/16 (31.25%) | 74/199 (37%) | 0.64 |
| 2DS2 | 6/16 (37.5%) | 102/199 (51%) | 0.29 |
| 2DS3 | 3/16 (18.75%) | 45/199 (23%) | 0.69 |
| 2DS4 | 16/16 (100%) | 189/199 (95%) | 0.36 |
| 2DS5 | 5/16 (31.25%) | 27/99 (27%) | 0.72 |
| 2DP1 | 16/16 (100%) | no data | - |
| 3DL1 | 16/16 (100%) | 186/199 (93%) | 0.27 |
| 3DL2 | 16/16 (100%) | 99/99 (100%) |  |
| 3DL3 | 16/16(100%) | no data | - |
| 3DS1 | 5/16 (31.25%) | 71/199 (36%) | 0.70 |
| 3DP1f2 | 16/16 (100%) | no data | - |
| 3DP1f1 | 2/16 (12.5%) | no data | - |

* allelefrequencies.net, Germany KIR and Germany Hesse KIR population, corrected for multiple tests

** based on Pearson Chi-square Test/ Fisher Exact Test, corrected for multiple tests

Additional File

Table 3: KIR genes and Haplotype regarding clinical outcome

| KIR  present | recovered | dead | p-value* |
| --- | --- | --- | --- |
| 2DL1 | 10/10 (100%) | 6/6 (100%) | - |
| 2DL2 | 3/10 (30 %) | 4/6 (66.7 %) | 0.16 |
| 2DL3 | 10/10 (100%) | 6/6 (100%) | - |
| 2DL4 | 10/10 (100%) | 6/6 (100%) | - |
| 2DL5 | 4/10 (40%) | 4/6 (66.7%) | 0.31 |
| 2DS1 | 3 /10 (30 %) | 2/10 (33.3 %) | 0.89 |
| 2DS2 | 5/10 (50%) | 1/6 (16.7%) | 0.19 |
| 2DS3 | 2/10 (20 %) | 1/6 (16.7 %) | 0.87 |
| 2DS4 | 10/10 (100%) | 6/6 (100%) | - |
| 2DS5 | 3/10 (30%) | 2/6 (33.3%) | 0.89 |
| 2DP1 | 10/10 (100%) | 6/6 (100%) | - |
| 3DL1 | 10/10 (100%) | 6/6 (100%) | - |
| 3DL2 | 10/10 (100%) | 6/6 (100%) | - |
| 3DL3 | 10/10 (100%) | 6/6 (100%) | - |
| 3DS1 | 3/10 (30%) | 2/6 (33.3%) | 0.89 |
| 3DP1f2 | 10/10 (100%) | 6/6 (100%) | - |
| 3DP1f1 | 2/10 (20%) | 0/6 (0%) | 0.25 |
| KIR Haplotype  A  B | 1/10 (10%)  9/10 (90%) | 1/6 (16.7%)  5/6 (83.3%) | 1.0 |

*based on Pearson Chi-square Test/Fisher Exact Test, corrected for multiple tests

Additional File:

Figure 1

Kaplan Meier Estimates on Recovery regarding KIR genotype. Cumulative estimates on recovery from COVID-19 related severe ARDS showing no statistically significant differences regarding specific KIR genotype.


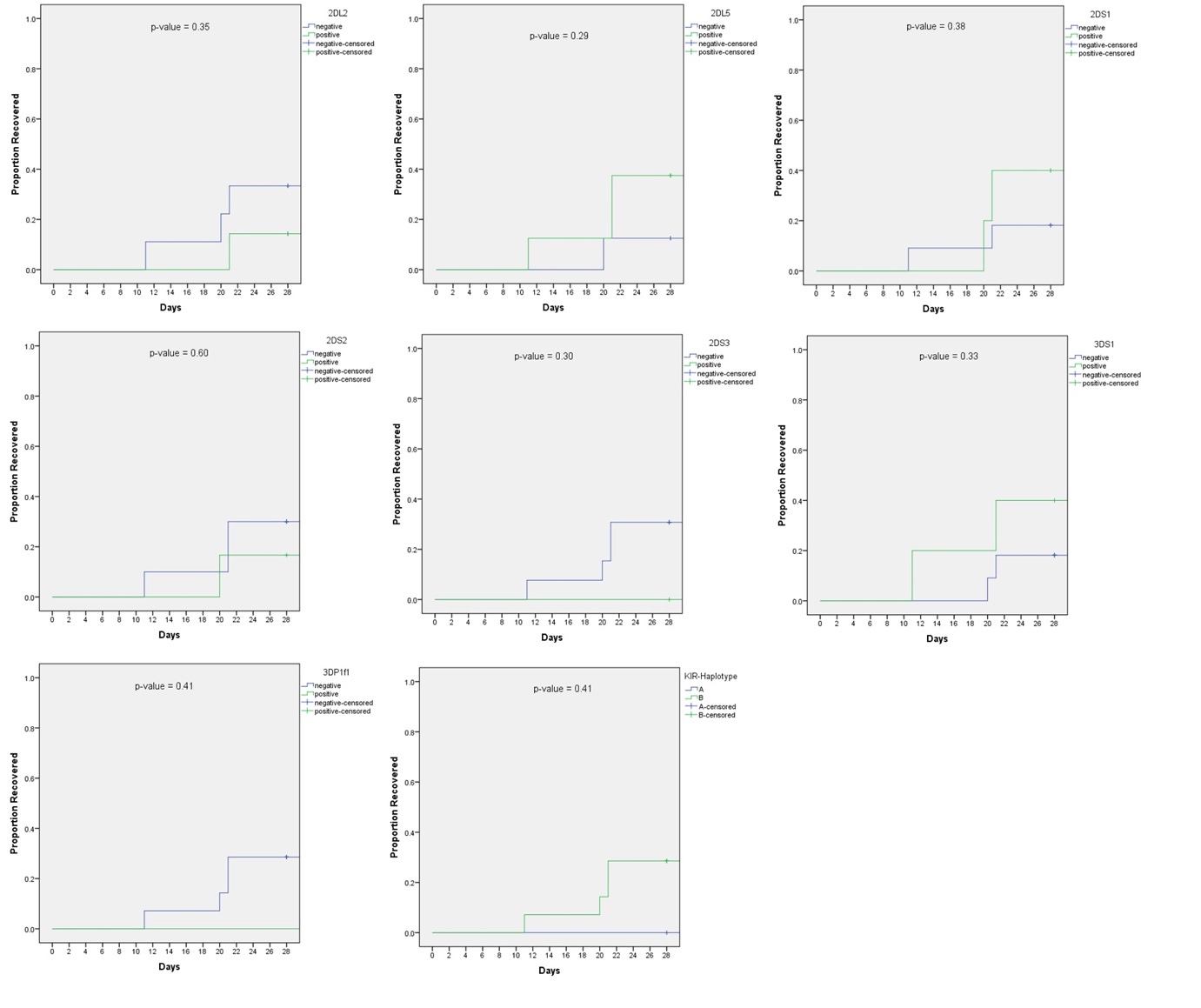


Additional File

Table 4. Clinical characteristics and laboratory parameters regarding KIR2DS5 status

| Parameters | KIR2DS5-positive  5 (100%) | KIR2DS5-negative  11 (100%) | p-value* |
| --- | --- | --- | --- |
| median age in years, (IQR) | 65 (11) | 60 (12) | 0.59 |
| baseline P/F ratio (IQR), mmHg | 97 (43) | 83 (23) | 1.0 |
| Invasive mechanical ventilation, % | 5(100) | 11 (100) | - |
| ECMO, % | 4(80) | 7(64) | 0.53 |
| complications during ICU stay, % | 1 (20) | 5 (45.5) | 0.58 |
| median time on ECMO in days, (IQR) | 20 (13) | 38 (43.25) | 1.0 |
| median WBC count at baseline (IQR), cells/µl | 10.000 (12755) | 9880 (6740) | 0.91 |
| median Lymphocyte count at baseline (IQR), cells/µl | 720 (998) | 588 (525) | 0.74 |
| median NK cell count at baseline (IQR), cells/µl | 58 (173) | 60 (58) | 0.66 |
| median CD4/CD8 ratio at baseline, (IQR) | 6 (12) | 3.6 (2) | 0.51 |
| median NK cell count during whole observational period (IQR), cells/µl | 96 (107) | 116 (94) | 0.34 |

* based on independent Median Test and Chi-square Test/Fisher’s Test, corrected for multiple tests

| Parameters | All patients  65 (100%) | KIR2DS5-negative  45 (100%) | KIR2DS5-positive  20 (100%) | p-value* |
| --- | --- | --- | --- | --- |
| Median Age in years, (IQR) | 68 (22) | 69 (23) | 68 (20) | 0.69 |
| Gender, %  Male  female | 37 (57)  28 (43) | 26 (58)  19 (42) | 11 (55)  9 (45) | 0.83 |
| Oxygen supplementation, % | 23 (35) | 19 (46) | 4 (22) | 0.08 |
| Median maximal CRP, mg/dl (IQR) | 94.5 (105) | 120 (115) | 83 (101) | 0.18 |
| ICU admission, % | 9 (13) | 9 (22) | 0(0) | 0.02 |
| Dead, % | 7 (10) | 6 (14) | 1(5) | 0.33 |

Additional File

Table 5. Baseline characteristics of 65 patients with moderate COVID-19 (validation cohort) according to KIR2DS5 status

* based on Pearson Chi-square Test with Yates Correction and Independent Median Test, both corrected for multiple comparison

Additional File:

Figure 2

Kaplan Meier Estimates on transfer to Intensive Care Unit (ICU) of 65 patients with moderate COVID-19 (validation cohort) according to KIR2DS5 Status. Cumulative estimates on time to transfer to intensive care unit (ICU) showing statistical significant difference regarding KIR2DS5 status.


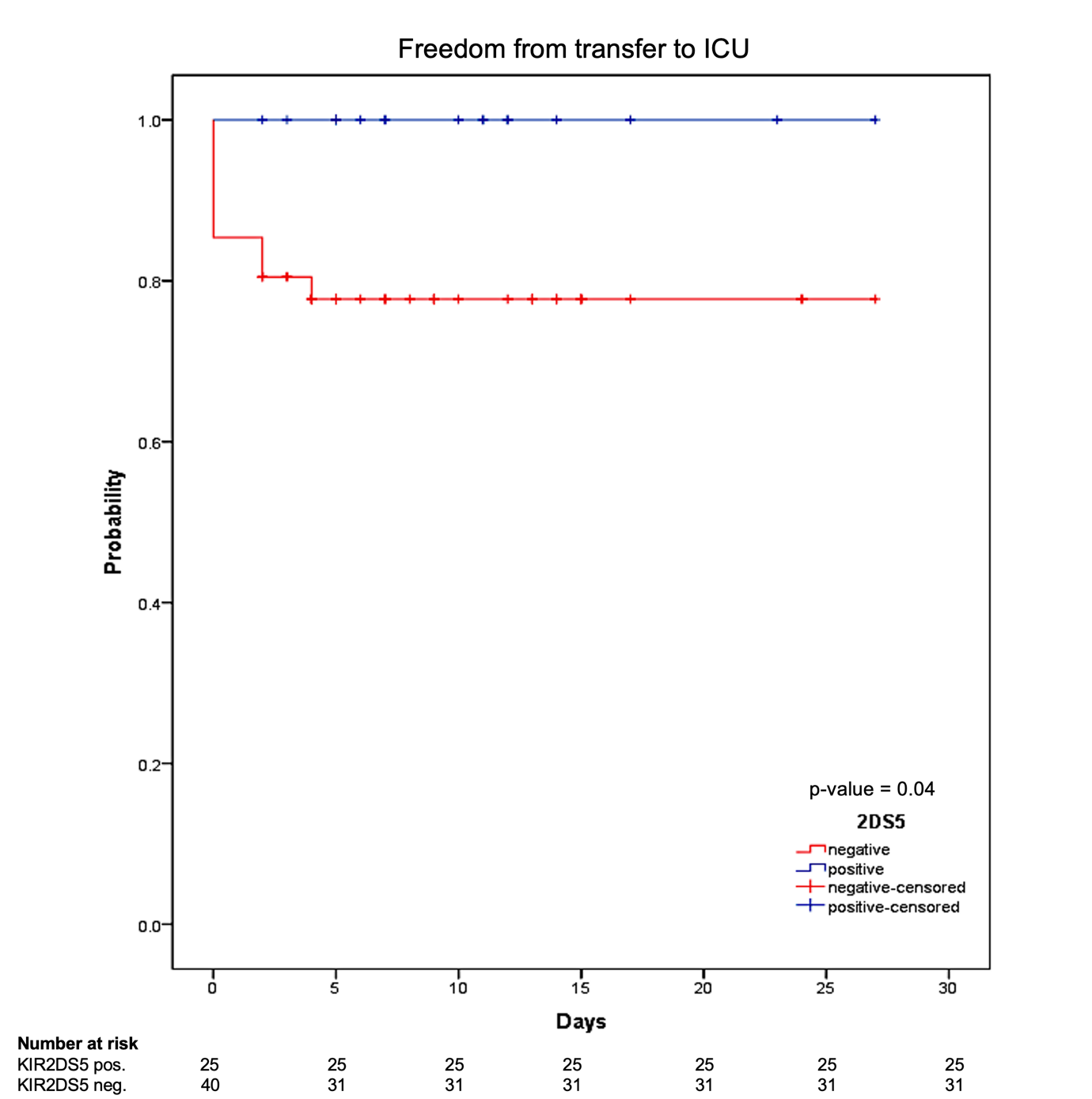

Supplement: Supplementary file 1 — Additional file 1: Table S1: Clinical characteristics and laboratory parameters based on recovery by day 28 (discovery cohort). Table S2: KIR prevalence in 16 patients with COVID-19 related severe ARDS compared to the general German Caucasian population. Table S3: KIR genes and Haplotype regarding clinical outcome. Figure S1. Kaplan Meier Estimates on Recovery regarding KIR genotype. Cumulative estimates on recovery from COVID-19 related severe ARDS showing no statistically significant differences regarding specific KIR genotype. Table S4. Clinical characteristics and laboratory parameters regarding KIR2DS5 status. Table S5. Baseline characteristics of 65 patients with moderate COVID-19 (validation cohort) according to KIR2DS5 status. Figure S2. Kaplan Meier Estimates on transfer to Intensive Care Unit (ICU) of 65 patients with moderate COVID-19 (validation cohort) according to KIR2DS5 Status. Cumulative estimates on time to transfer to intensive care unit (ICU) showing statistical significant difference regarding KIR2DS5 status. [file 40635_2021_409_MOESM1_ESM.docx]
